# Rational combination of MEK inhibitor and the STAT3 pathway modulator for the therapy in K-Ras mutated pancreatic and colon cancer cells

**DOI:** 10.18632/oncotarget.3991

**Published:** 2015-05-04

**Authors:** Chengguang Zhao, Hui Xiao, Xiaojuan Wu, Chenglong Li, Guang Liang, Shulin Yang, Jiayuh Lin

**Affiliations:** 1 School of Environmental and Biological Engineering, Nanjing University of Science and Technology, Nanjing, Jiangsu, People's Republic of China; 2 Center for Childhood Cancer and Blood Diseases, The Research Institute at Nationwide Children's Hospital, Department of Pediatrics, College of Medicine, The Ohio State University, Columbus, OH, USA; 3 Chemical Biology Research Center, School of Pharmaceutical Sciences, Wenzhou Medical University, University Town, Wenzhou, Zhejiang, People's Republic of China; 4 Division of Medicinal Chemistry and Pharmacognosy, College of Pharmacy, The Ohio State University, Columbus, OH, USA

**Keywords:** STAT3, K-Ras, MEK inhibitor, pancreatic cancer, colon cancer

## Abstract

K-Ras mutations are frequently detected in pancreatic and colon cancers, which are associated with the resistance to MEK inhibitors targeting the Ras pathway. Identifying the underlying mechanisms for the acquired resistance is essential for the future clinical development of MEK inhibitors. Here, we identified that Signal Transducer and Activator of Transcription 3 (STAT3) was significantly activated following the MEK inhibition using AZD6244, PD98059 and Trametinib in K-Ras mutant pancreatic and colon cancer cells. The STAT3 activation may be important for the MEK inhibitor resistance in these K-Ras mutant cancer cells. We have shown that dual inhibition of STAT3 and MEK using the STAT3 inhibitor LY5 and MEK inhibitor Trametinib exerts significant anti-tumor cell efficacy in K-Ras mutant pancreatic and colon cancer cells *in vitro*. In addition, Trametinib showed increased suppression on tumor growth *in vivo* in STAT3 knockdown pancreatic cancer cells compared with tumor growth of control cells without STAT3 knockdown. Taken together, our results suggest the induced STAT3 activation as a possible mechanism for the resistance to MEK inhibitor and demonstrate the potentials of a combination therapy using MEK and STAT3 inhibitors in pancreatic and colon cancers harboring K-Ras mutant proteins.

## INTRODUCTION

Ras proteins play a direct causal role in human cancers. Oncogenic mutant Ras proteins are highly prevalent in multiple human tumors (~30% of all human tumors), with mutations of K-Ras being the major clinical problem [1]. Oncogenic K-Ras mutations occur in 90% of pancreatic and 45% of colorectal carcinomas [2], and these mutations are associated with the resistance to MEK inhibitors [3, 4]. K-Ras mutations in cancers are localized frequently at codon 12 and much less commonly at codon 13, 17, 34, or 61; COSMIC (Catalogue of Somatic Mutations in Cancer) database [5]. In pancreatic cancer, mutations are essentially seen only at the codon 12, with rare exceptions at codon 13 [5-7]. Mutations at codon 12 are commonly found of substitution of glycine with aspartic acid (G12D), valine (G12V), cysteine (G12C) or others [8] resulting in a conformational change of a protein site located near the bound nucleotide (GTP) and leads to constitutive activation of the mutated Ras proteins independently of growth factor stimulation. In colon cancer, the most frequent mutations in K-Ras are guanine to adenine transitions and guanine to thymine-conversion with 90% of the somatic point mutations occurring at hotspot codon 12 (G12D, 70%) or 13 (G13D, 30%) in exon 1 [9-11]. Moreover, cancers with a high prevalence of K-Ras mutations, such as pancreatic carcinomas, colorectal cancers and lung cancers are difficult to treat. Clinical outcomes are poor even with aggressive and toxic medical interventions [12, 13]. Suppressing K-Ras mutants has become a promising concept for new therapies. Recently, inhibitors of K-Ras mutants have been developed and tested to show some activity in cancer cell lines and tumor models [14, 15]. However, K-Ras mutant have been proven highly difficult to drug, and no small molecular K-Ras mutant inhibitors are available for clinical trials yet. This places the K-Ras mutants target in the so-called difficult-to-drug target category [16, 17]. With the failure of directly inhibiting K-Ras mutant, inhibiting downstream effectors of K-Ras appears a promising alternative [18]. K-Ras signals via downstream effectors such as MAPK, PI3K/AKT and STAT3 signaling cascade [19-21]. It has been shown MAPK signaling plays a more important role in tumor maintenance than PI3K signaling in K-Ras mutant pancreatic and lung tumors [22, 23]. Drug development efforts have mostly focused on components of the classical Ras-activated MAPK pathway. As part of this pathway, MEK1/2, a dual-specific kinase required for activation of ERK1/2, plays crucial roles in tumorigenesis, cell proliferation and inhibition of apoptosis, therefore, MEK1/2 inhibition is an attractive therapeutic strategy in a number of cancers. Inhibiting the downstream effector MEK1/2 has proven to be effective in preclinical studies. Several MEK inhibitors (AZD6244, trametinib and others) have been developed and are under investigation in clinical studies in colon cancer [24-26] and pancreatic cancer [27, 28]. MEK inhibitors have the potential to inhibit tumors dependent on MAPK signaling pathway. Unfortunately, drug resistance limits their efficacy in some patients. Resistance to MEK inhibitors has been reported in pancreatic cancer and colon carcinoma with Ras mutations. However, the underlying mechanism is not very clear. Elucidating the mechanism of cancer cell resistance to MEK inhibitors is critical for the development of more effective therapies.

Our preliminary results have shown that the MEK inhibition in K-Ras mutant pancreatic cancer cells and colon cancer cells unexpectedly induced STAT3 phosphorylation/activation. STAT3 is an oncogene, which is constitutively activated in multiple types of human cancers and contributes to cancer progression [29, 30]. STAT3 activation can stimulate oncogenic transformation in cultured cells and tumor formation in nude mice [31]. In contrast, STAT3 deficient fibroblasts are resistant to transformation by a variety of oncogenes [32, 33]. STAT3 is activated (persistent phosphorylation of STAT3) in many human pancreatic [19, 34, 35] and colon cancer cells [36-41]. However, STAT3 is not persistently active in normal pancreatic tissues and not required for pancreatic development or homeostasis [42]. Activation of STAT3 is also a marker of poor prognosis in human colon cancer [43]. Persistent STAT3 signaling is an attractive target due to its role in regulating cell proliferation, apoptosis, invasion, angiogenesis and immune suppression [29]. Furthermore, STAT3 inhibition can promote apoptosis of human pancreatic [44-48] and colon cancer cells [36, 38, 49] *in vitro* and *in vivo*. Since STAT3 phosphorylation/activation can be induced by MEK inhibitors, we hypothesize that the combined treatment with STAT3 and MEK inhibitors would have enhanced therapeutic effects in K-Ras mutant pancreatic cancer and colon cancer cells compared with MEK or STAT3 inhibitor alone.

In this study, we observed that STAT3 pathway activation is a hallmark response to MEK inhibition in K-Ras mutated cells, and found that coinhibition of STAT3 and MEK mediators could overcome this resistance. Phosphorylation and activation of STAT3 can also occur upstream upon the activation of Janus kinases (JAKs). We further assessed the anti-tumor effects of JAK2/STAT3 inhibitor and MEK inhibitor as single agents and in various combinations in K-Ras mutated pancreatic and colon cells *in vitro*, as well as nude mice tumor model *in vivo*. The results may provide a basis for further development of novel therapeutic strategies for the treatment of K-Ras mutant cancer.

## RESULTS

### Increased STAT3 and JAK2 phosphorylation induced by MEK inhibitors in K-Ras mutant pancreatic cancer cells

**Figure 1:**
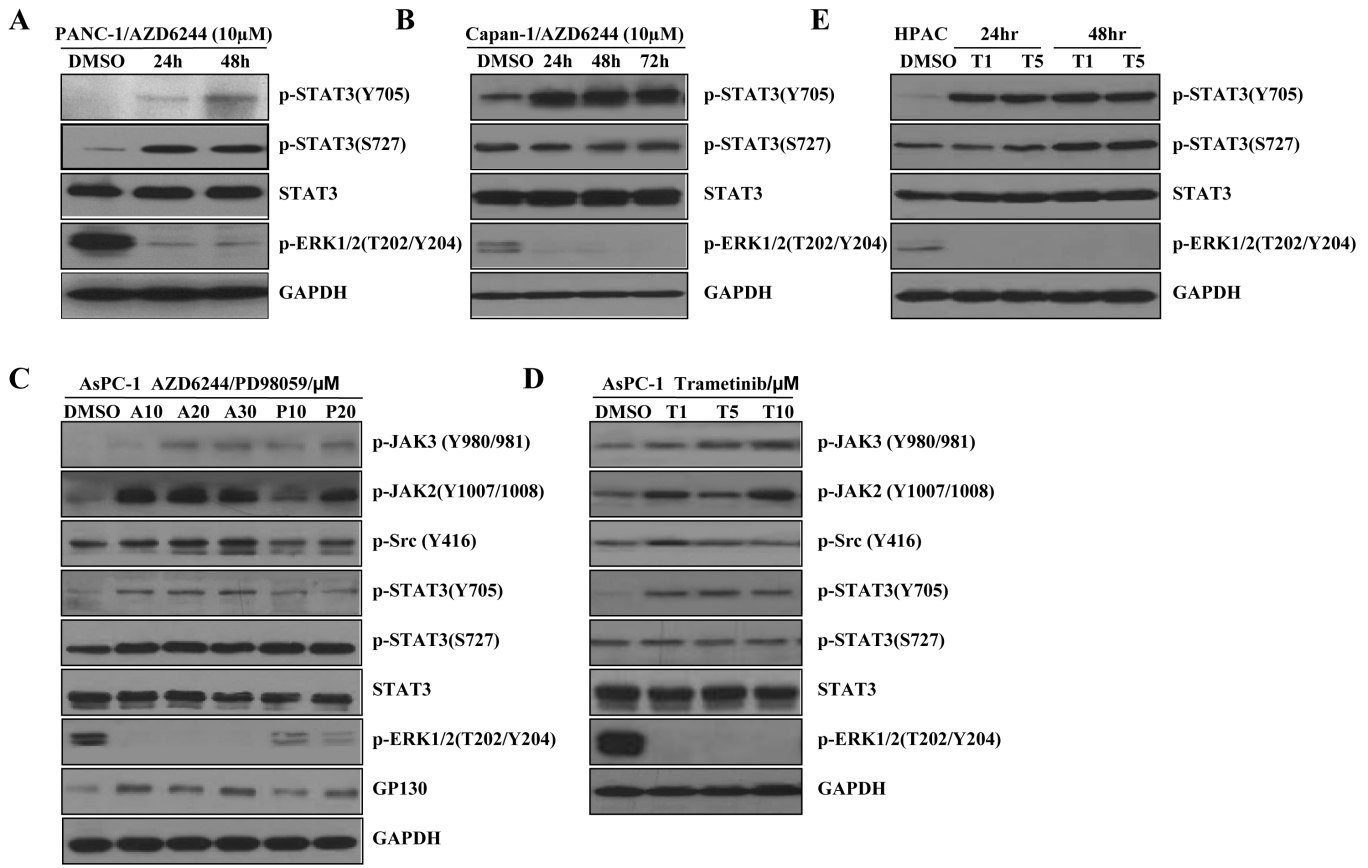
MEK inhibitors induce P-STAT3 in K-Ras mutant pancreatic cancer cells (**A**) PANC-1 cancer cells were treated with AZD6244 (10 μM). After 24 and 48 hr, cell lysates were subjected to western blot with the indicated antibodies. GAPDH served as a loading control. (**B**) Capan-1 cancer cells were treated with AZD6244 (10 μM). After 24, 48 and 72 hr, total cell protein extracts (60 mg) were subjected to immunoblotting with the indicated antibodies, as described in the materials and methods. GAPDH was a loading control. (**C**) AsPC-1 cancer cells were treated with or without MEK inhibitors at the indicated dose for 24 hr, and P-JAK3, P-JAK2, P-Src, P-STAT3(Y705), P-STAT3(S727), STAT3, P-ERK1/2, and GP130 levels were determined by western blot. GAPDH was a loading control. (**D**) AsPC-1 cells were treated with trametinib (1, 5, 10 μM) for 24 hr. The indicated proteins were detected by western blot. GAPDH served as a loading control. (A: AZD6244; P: PD98059; T: trametinib). (**E**) HPAC cells were treated with trametinib (1, 5μM) for 24 or 48 hr. The indicated proteins were detected by western blot.

MEK inhibitor, AZD6244, showed activity in K-Ras mutant advanced non-small cell lung cancer in clinical trials [50] and is being investigated for pancreatic cancer therapy in phase II clinical trials [28]. To investigate whether MEK inhibitors activate JAK2/STAT3 signal pathways, we examined the effects of MEK inhibitors in K-Ras mutant cancer cell lines. First, we tested the effects of AZD6244 in K-Ras mutant pancreatic cancer cells. Pancreatic cancer cell lines PANC-1 (G12D) and Capan-1 (G12V) were treated with 10 μM of AZD6244 for 24-72 hours. As expected, AZD6244 effectively inhibited phosphorylated ERK1/2 (P-ERK1/2) in the two cell lines (Figure 1A and 1B). Interestingly, AZD6244 induced STAT3 Tyr705 phosphorylation, P-STAT3 (Y705), in both PANC-1 and Capan-1 cells at the different time point, but it did not increase P-STAT3(S727) in Capan-1 cell (Figure 1A and 1B). To further determine whether STAT3 activation was associated with MEK inhibition, we tested another small molecule MEK inhibitor PD98059 [51] in a different K-Ras mutant pancreatic cancer cell line, AsPC-1 (G12D). As anticipated, PD98059 increased P-STAT3 (Y705) and P-STAT3 (S727) in AsPC-1 (G12D) cells. However, P-STAT3 (S727) was not markedly increased (Figure 1C). AZD6244 and PD98059 are not approved for clinical use by the FDA. Trametinib, another MEK inhibitor, has been approved for treating melanoma [52, 53]. So, we tested trametinib in the AsPC-1 (G12D) and HPAC (G12D) cells. Similarly, P-STAT3(Y705) was significantly increased in AsPC-1 and HPAC cells after 24 or 48 hours of the treatment with trametinib at different concentrations, while P-STAT3 (S727) was not markedly increased (Figure 1D and 1E). Here, remarkable increases in P-STAT3(Y705) upon MEK inhibition were observed in all four pancreatic cancer cell lines (Figure 1) whereas, slight increases in P-STAT3(S727) were observed in three cancer cell lines (Figure 1A, 1C and 1E). In the Capan-1(Figure 1B) and AsPC-1(Figure 1D) cells, P-STAT3(S727) was virtually unchanged or decreased. These results suggest that activation of STAT3 upon MEK inhibition is mainly by phosphorylation of Tyr705 residue.

Src family kinases have been found to activate STAT3 and linked to drugs resistance in some cell lines and patient samples [54]. We therefore examined the activation of the Src proteins in K-Ras mutant pancreatic cancer cells (AsPC-1 as a representative). In AsPC-1 cells with the treatment of AZD6244, PD98059 or trametinib resulted in a slight increase of P-Src (Y416) (Figure 1C and 1D), suggesting the activation of P-STAT3 may not be dependent on Src family kinases. JAK and GP130 are direct upstream effectors of STAT3. Overexpression of JAK and GP130 can directly activate STAT3 [55]. We also assessed the effect of MEK inhibitors on signal transduction involving the IL6-GP130/JAK/STAT3 pathway. JAK3 phosphorylation and GP130 were moderately increased. Interestingly, P-JAK2 was significantly induced in all investigated pancreatic cancer cell lines (Figure 1C and 1D). Taken together, these results suggest that JAK2 may be the main upstream kinase that activates the STAT3 signaling in response to MEK inhibitors.

### Increased STAT3 phosphorylation induced by MEK inhibition in K-Ras mutant colon cancer cells

**Figure 2:**
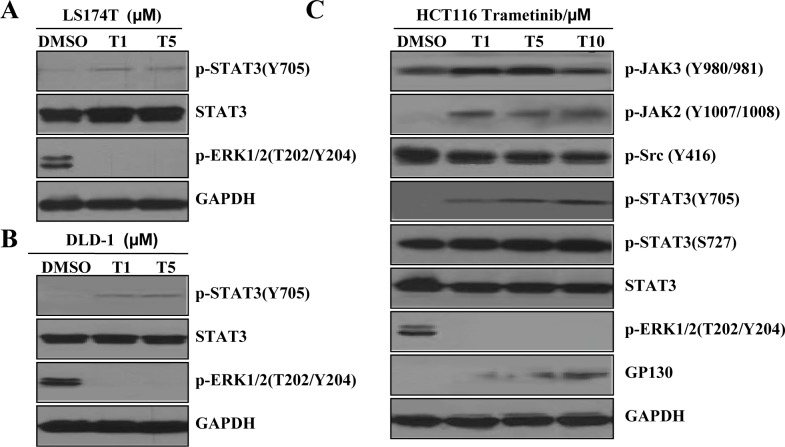
MEK inhibition induces STAT3 phosphorylation in K-Ras mutant colon cancer cells (**A**) LS174T cells were treated with 1 and 5 μM trametinib (T) for 72 hr. P-STAT3 (Y705), STAT3 and P-ERK1/2 expression and activity levels were determined by western blotting. (**B**) DLD-1 cells were treated with 1 and 5 μM trametinib (T) for 72 hr. P-STAT3 (Y705), STAT3 and P-ERK1/2 expression and activity levels were determined by western blotting. (**C**) HCT116 cells were treated with 1, 5, 10 μM trametinib (T) for 24 hr. Proteins expression and activity levels were determined by western blotting. GAPDH served as the loading control.

Trametinib inhibited phosphorylated ERK1/2 (P-ERK1/2) in K-Ras mutant colon cancer cell lines HCT116 (G13D), LS174T (G12D) and DLD-1 (G13D) (Figure 2). Surprisingly, trametinib also induced phosphorylation of STAT3 at Tyr705 (P-STAT3 Y705), but not P-STAT3 (S727). Trametinib decreased ERK phosphorylation in K-Ras mutant colon cancer cells, while it also induced a strong expression of P-JAK2 and a moderate expression of P-JAK3, the upstream kinases that phosphorylate STAT3 (HCT116 cells as a representative, Figure 2C). However we did not observe increases in Src phosphorylation in K-Ras mutant colon cancer cells. Our novel results suggest that the activated/phosphorylated Tyr705 of STAT3 (P-STAT3 Y705) promotes cancer cell survival and causes feed-back resistance to MEK inhibitors.

### JAK2/STAT3 activation is key mediators of resistance to MEK inhibitors in K-Ras mutant cells

**Figure 3:**
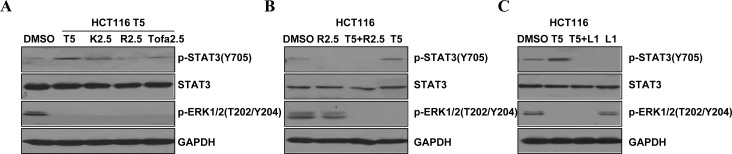
Efficacy of JAK2 and STAT3 inhibitors in K-Ras mutant HCT116 cells to block feedback-induced phosphorylation of STAT3 induced by MEK inhibitor (**A**) Western blot (WB) of phosphorylated STAT3 (P-STAT3), phosphorylated ERK (P-ERK), total STAT3 and GAPDH in HCT116 K-Ras mutant (G13D) cells after overnight treatment with different inhibitors (T: trametinib, K: KX2-391, R: ruxolitinib, Tofa: tofacitinib). (**B**) HCT116 cells were treated with trametinib (5 μM) alone or in combination with ruxolitinib (2.5 μM) for 24 hr, and P-STAT3(Y705), STAT3 and P-ERK1/2 was determined by WB. (**C**) HCT116 cells were treatment with trametinib (5 μM) or combined LY5 for 24 hr, and P-STAT3(Y705), STAT3 and P-ERK1/2 levels were determined by WB. GAPDH served as a loading control.

Activation of STAT3 can be through one of these kinases, JAK2, JAK3 or the Src family kinases. To explore the molecular significance and therapeutic implications of mutations in the K-Ras pathway, we also assessed the phosphorylation of ERK and STAT3 after treatment with those kinases inhibitors. Cancer cell line HCT116 was treated with trametinib (5μM) alone, or in combination with one of the following agents: ruxolitinib (2.5μM, JAK2 inhibitor), tofacitinib (2.5μM, JAK3 inhibitor) or KX2-391 (2.5μM, Src inhibitor). Under these conditions, ERK phosphorylation was inhibited in the presence of trametinib (Figure 3). However, STAT3 phosphorylation was also induced by trametinib along with the MEK inhibition in the HCT116 cells. Expectedly, increased STAT3 phosphorylation induced by trametinib was down regulated by ruxolitinib (JAK2) inhibition, and partially by tofacitinib (JAK3) inhibition. But the phosphorylation of STAT3 was not decreased by the inhibition of Src family kinases in the cell lines (Figure 3A). These results suggest that the trametinib-induced phosphorylation of STAT3 may through the JAK2/STAT3 signal transduction pathway.

We also performed immunoblots to gauge the effects of a combination of MEK inhibitor and JAK2 inhibitor in the K-Ras mutant HCT116 cell lines (Figure 3B). When cells were treated with trametinib in combination with ruxolitinib (JAK2 inhibitor), the trametinib induced activation of STAT3 was inhibited. Given the promising response of HCT116 cells to the dual inhibition of the JAK2/STAT3 and MEK/ERK pathways, we further investigated the effects of a combination of the STAT3-selective inhibitor LY5 and the MEK inhibitor trametinib in K-Ras mutant cell lines (Figure 3C). The combined treatment of LY5 and trametinib also synergistically inhibited ERK phosphorylation and STAT3 activation induced by trametinib in the HCT116 cell line. These findings provide additional evidence for that STAT3 activation in response to MEK inhibition mediates the antagonistic effects of the MEK inhibitors in K-Ras mutant colon cells.

### Effect of the expression of STAT3 protein on MEK inhibition

**Figure 4:**
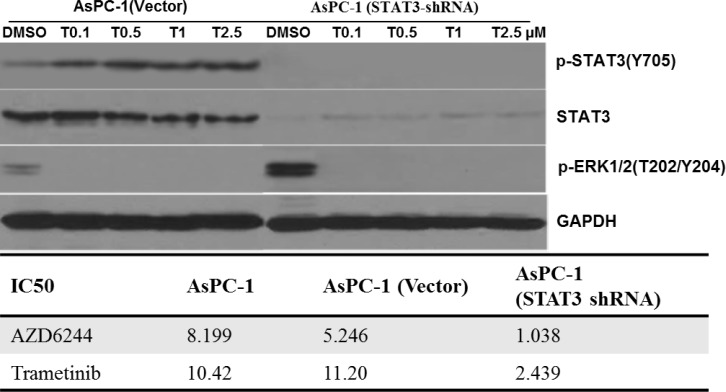
Knocking down STAT3 expression using RNA interference, resulted in enhanced sensitivity to MEK inhibitors AsPC-1 cells were infected with shRNA against STAT3 and non-targeted shRNA (Vector). Those cancer cell lines were treated with trametinib at the indicated dose for 24 hr. The expressions of P-STAT3, P-ERK1/2 and STAT3 were detected on western blots, GAPDH served as a loading control. IC_50_ was determined as described in the materials and methods.

To confirm the essential role of the STAT3 pathway in resistance to MEK inhibition, we knocked down STAT3 in K-Ras mutant AsPC-1 cell line. In cell viability assays, knockdown of STAT3 in the AsPC-1 cells resulted in enhanced sensitivity to AZD6244/trametinib. The knockdown cell lines, AsPC-1 (STAT3-shRNA), showed that the half-maximum inhibitory concentration (IC_50_) were at least 5-fold lower than their parental cells (AsPC-1) and control cells (AsPC-1, vector) (Figure 4). MEK inhibition had small effects in the parental cells AsPC-1 and the control AsPC-1(vector), while it had significant effects on inhibiting cell viability of the AsPC-1(STAT3-shRNA) cell lines. These results suggest that activation of STAT3 stimulated by MEK inhibition is necessary for cancer cell survival. Therefore, STAT3's inhibition may be a potential strategy for overcoming the MEK inhibitor resistance. We then examined the expression levels and phosphorylation of STAT3 protein after MEK inhibition. In control cells AsPC-1 (vector), increasing doses of trametinib for 24 hours caused a decrease in P-ERK, but an increase of P-STAT3 (Y705). In AsPC-1 (STAT3-shRNA), P-ERK was also inhibited by the treatments (Figure 4), but no P-STAT3 (Y705) was detected.

**Figure 5:**
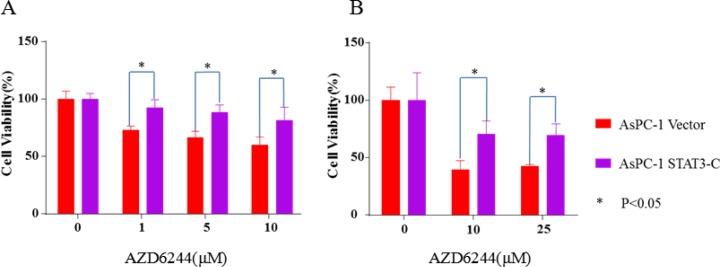
Constitutively active STAT3, STAT3-C reversed the inhibition induced by AZD6244 in AsPC-1 cells Cloned AsPC-1 cells with or without STAT3-C expression vector, followed by AZD6244 treatment at the indicated concentration for 24 (**A**) or 48 (**B**) hr. MTT assay was used to analyze cell viability. Error bars indicate SD of mean.

To further validate our hypothesis and verify that the effect of resistance to MEK inhibitors is correlated to STAT3 activation, we stably express a known constitutively active STAT3, STAT3-C [31], in cancer cells. The inhibition of cell viability of AZD6244 in AsPC-1 cells was also partially reversed by the transfection with STAT3-C expression (Figure 5). AsPC-1 (Vector) cells not transferred with STAT3-C are still relatively sensitive to AZD6244 inhibition. Our results show that STAT3-C can at least partially rescue MEK inhibition. These results provide additional evidence to support that the resistance to MEK inhibitors is through the induction of endogenous STAT3 protein in cancer cells. Collectively, knockdown of STAT3 in AsPC-1 cells significantly sensitized the cells to AZD6244 and trametinib treatment, and activation of the STAT3 pathway by the constitutively active STAT3 induced the resistance to AZD6244. These results suggest that STAT3 pathway activation may play an important role in developing resistance to MEK inhibitors.

### Combinational inhibition of MEK and STAT3 pathways in K-Ras mutant pancreatic and colon cancer cells

Biochemical feedback loops and cross-talk may drive primary resistance to MEK-inhibitors [56, 57]. This provides a rationale for combining MEK-inhibitors with Raf, PI3K, FGFR or EGFR inhibitors [58]. Since STAT3 signaling is involved in cell survival and drug resistance [10, 59-61], the undesired STAT3 activation by MEK inhibitors could potentially introduce resistance to MEK inhibitors in clinical trials. Therefore, this novel finding provides a rationale for a combination of MEK inhibitors and STAT3 pathway modulators to afford more effective therapy of K-Ras mutant cancer.

**Figure 6:**
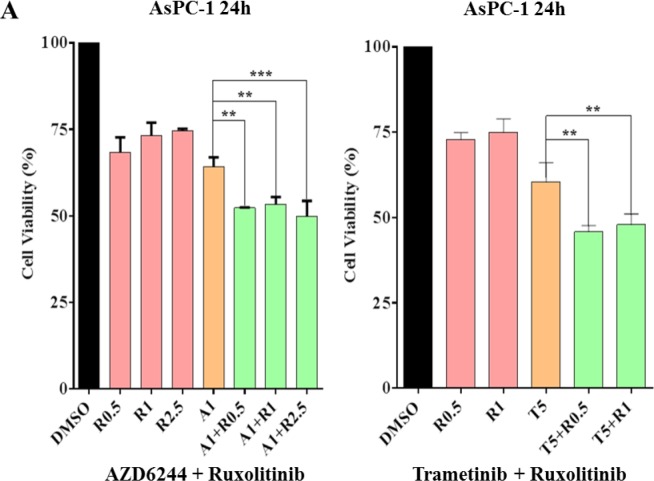

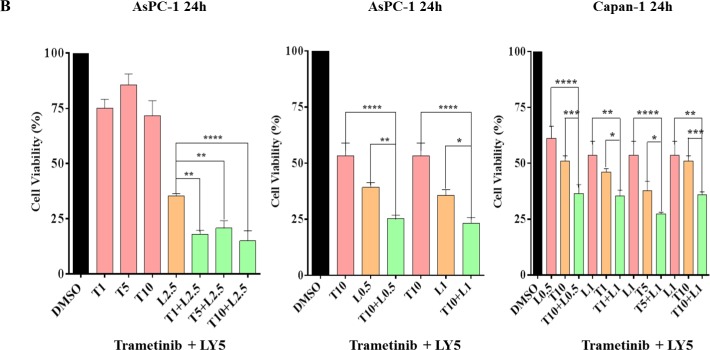

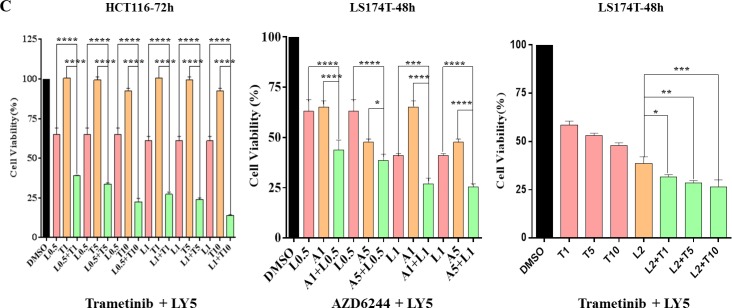
Combinational treatment decrease viability of K-Ras mutant cancer cell lines (**A**) AsPC-1 cells were seeded in 96-well plate at a density of 3000 cells per well and cultured for 24 hr. Cells were treated with trametinib/AZD6244 or/and ruxolitinib at the indicated concentration in triplicate for 24 hr and processed for MTT assay to analyze cell viability. (**B**) AsPC-1 and Capan-1 cells were seeded the same way as described in A. Cells were treated with trametinib or/and LY5 at the indicated concentration in triplicate for 24 hr to detect the combination effects of LY5 and trametinib using MTT assay. (**C**) HCT116 and LS174T cells were seeded the same as described in A. Cells were treated by trametinib/AZD6244 or/and LY5 at the indicated concentration in triplicate for 48 or 72 hours to detect the combination effects of LY5 and trametinib using MTT assay. Error bars indicate SD of mean. (* *P* < 0.05, ** *P* < 0.01, *** *P* < 0.001, **** *P*< 0.0001).

Because JAK2 coordinately mediate MEK inhibitors that induced P-STAT3, this induction was inhibited by combinational treatment of ruxolitinib or LY5 as detected by western blot analysis (Figure 3). To examine whether the resistance can be reversed by JAK2/STAT3 inhibition, combination of several small molecular inhibitors were investigated to inhibit both the MEK and STAT3 pathways. AsPC-1 cell viability assays were assessed upon exposure to the different agents alone and in combination. As shown in Figure 6A, AsPC-1 cell viability was remarkably reduced with the combination of MEK inhibitor (AZD6244) and JAK1/2 inhibitor (ruxolitinib). In contrast, MEK inhibitor or JAK1/2 inhibitor as a single agent was not very effective on cancer cell viability. Similar results were obtained by co-treatment with trametinib and ruxolitinib (Figure 6A). The synergistic effects were also obtained by the combined treatment of LY5 and trametinib in AsPC-1 and Capan-1 cells (Figure 6B). The rationale to choose LY5 in our studies is that LY5 is a potent STAT3-selective small molecular inhibitor. LY5 alone (or knockdown STAT3) did not inhibit P-ERK1/2 but appeared to slightly induce P-ERK1/2 (Figure 4), and AZD6244 or trametinib alone, as well as the combination blocked P-ERK1/2. Similar results were observed with a combination of MEK and STAT3 inhibitors in K-Ras mutant HCT-116 and LS174T colorectal cancer cells (Figure 6C), which further support the hypothesis that inhibiting dual MEK and STAT3 pathways in K-Ras mutant cells can increase sensitivity to MEK inhibitors.

### Combinational inhibition of MEK and STAT3 pathways reduces colony forming and cell migration ability

**Figure 7:**
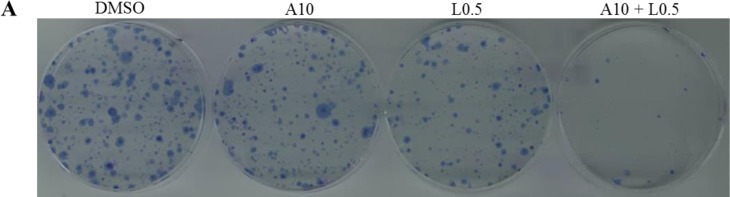

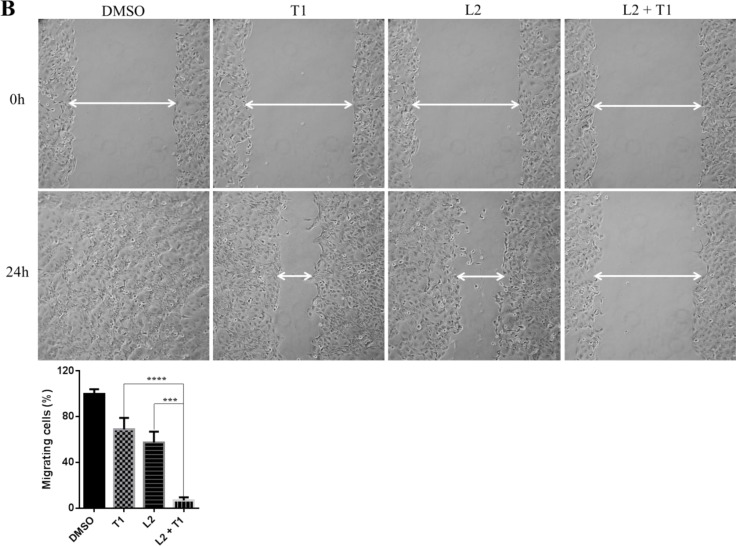

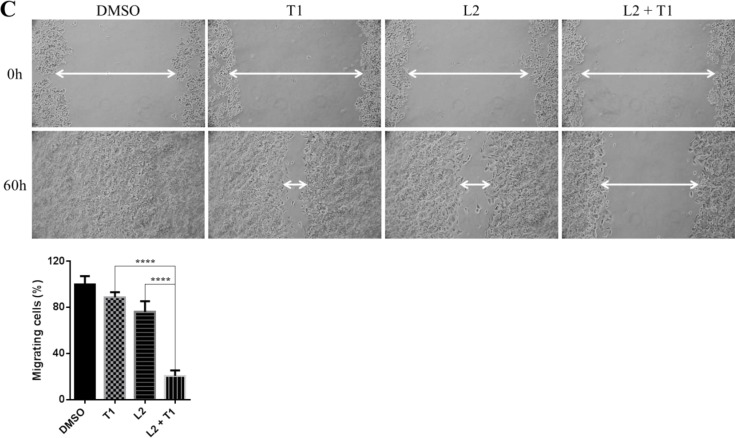

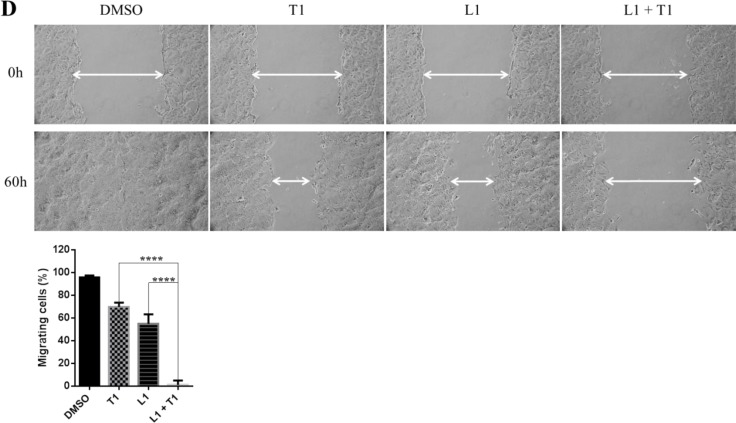
Combinational inhibition of MEK and STAT3 pathways reduces colony forming ability and cell migration (**A**) AsPC-1 cells were treated with drugs (A10: AZD6244 10μM, L0.5: LY5 0.5μM) or DMSO for 2 hours. After the treatment, cells were counted and the same number of cells were seeded and cultured for 2-3 week. Colonies were fixed by ice-cold methanol and were stained by 1% crystal violet. (**B**) Wound healing assay for migration was carried out by scratching the cells with yellow tip when HPAC grew into monolayer. Then, cells were treated with trametinib, LY5 or the combination of the two and allowed to migrate into the scratched area for 24 hours (T1: trametinib 1μM, L2: LY5 2μM). (**C**) Wound healing assay as described in materials and methods was conducted for migration in HCT116 cells treated with the indicated concentrations of inhibitors (T1: trametinib 1μM, L2: LY5 2μM). After 12 hours, growth medium was replaced with a fresh complete DMEM medium with 10% FBS. (**D**) Wound healing assay as described in C was conducted for migration in DLD-1 (T1: trametinib 1μM, L1: LY5 1μM). The arrow showed the gap of scratched area and the percentage of migrating cells in wound healing assay was quantified. Inset 100 x magnification.

We found that the combination of STAT3 and MEK inhibitors enhanced inhibition of cell viability compared with the treatment of a single agent in all tested cell lines (Figure 6). Blocking STAT3 activation by a combination of STAT3 inhibition (ruxolitinib or LY5) and MEK inhibition, showed a synergistic antitumor effect in K-Ras mutant cell lines. Therefore, we next sought to investigate whether combination treatment may also inhibit the colony formation capability. AsPC-1 cells were treated with AZD6244, LY5 or the combination of the two for 2 hours. After the treatment, the same number of viable cancer cells was seeded at very low cell densities (1000 cells/60mm dish) and cultured in fresh medium without drugs for 2 weeks. Cells were then fixed and stained and the plates were scanned. As shown in Figure 7A, the cancer cells showed a decreased ability to recover and form colonies following combination treatment with AZD6244 and LY5. The results demonstrate that the combination treatment exhibited stronger inhibition of the colony forming ability than a single agent, and indicate the benefit and rationale to block both MEK and STAT3 oncogenic pathways in K-Ras mutant pancreatic cancer cells.

Cell migration is an important process in tumor metastasis. In order to assess the effect of a dual inhibition of MEK and STAT3 pathways on cell migratory ability, a wound healing assay was performed on HPAC, HCT116 and DLD-1 cells. As shown in Figure 7B, after the creation of a wound, cells were treated with different inhibitors and allowed to grow and migrate into the wound for 24 hours. HPAC cells migrated to fill the scratched area within 24 hours. Treatment with LY5 or trametinib also resulted in a decreased ability for cells to migrate and heal the created wound, but the combined treatment of trametinib and LY5 completely prevented this migration. Consistent with above result, co-treatment also significantly blocked HCT116 and DLD-1 cells to migrate through scratched area (Figure 7C and 7D).

### Co-inhibition of the STAT3 and MEK signaling pathways inhibits the tumor growth of K-Ras mutant xenograft models

**Figure 8:**
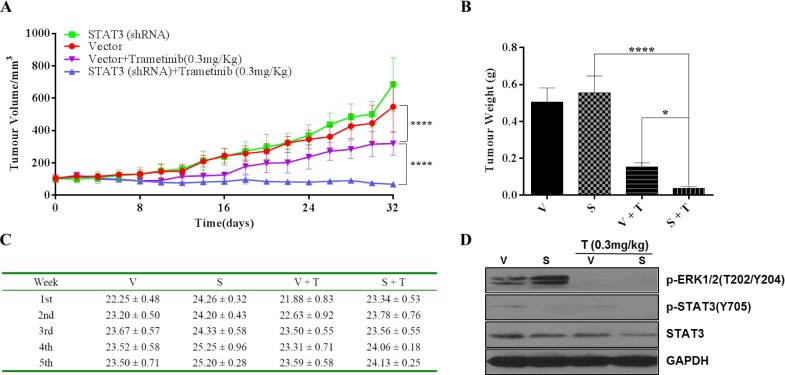
Anti-tumor effects of dual inhibition of STAT3 and MEK signaling in AsPC-1 xenograft model, tumor growth is shown Mice bearing AsPC-1-vector (V) and AsPC-1 STAT3-shRNA (S) tumors were treated with trametinib for 32 days. (A) Tumor volumes (mm^3^) and (B) Tumor weights (g) were recorded. Error bars indicate SD of mean. (C) Body weight of each mice was recorded. (D) P-ERK1/2, P-STAT3 and total STAT3 was measured in the isolated tumor samples by western blot, GAPDH served as a loading control. (T: trametinib, * *P* <0.05, **** *P* < 0.0001).

To determine the impact of STAT3 inhibition on tumor growth *in vivo*, we subcutaneously injected AsPC-1(STAT3-shRNA) and AsPC-1(Vector) cells into nude mice on either side of the abdomen. Mice were then treated with trametinib by daily oral gavage. Knockdown STAT3 (STAT3-shRNA) did not reduce the size of xenograft tumors compared with AsPC-1(Vector) group. In contrast, simultaneous inhibition of MEK and STAT3 (S + T) significantly reduced tumor growth compared to those control groups (Figure 8A curve in blue). Interestingly, in the (S + T) group with a dual inhibition, tumors appeared to grow slightly at the beginning, but remained essentially static over the 5 weeks' treatment. Although trametinib-treated AsPC-1(Vector) xenografts (V + T) impaired tumor growth, its effect was not stronger than in AsPC-1(STAT3-shRNA) xenografts (S + T) mice (Figure 8A and 8B). The result suggests that MEK or STAT3 signaling pathway alone is able to maintain proliferation. Inhibiting only one of the MEK and STAT3 signaling pathways would not be effective. Therefore, *in vivo*, a dual inhibition of MEK and STAT3 signaling pathways would be much more effective in K-Ras mutant cancer cells. Taken together, both *in vitro* and *in vivo* results suggest that STAT3 plays a critical role in K-Ras mutant cells in response to agents inhibiting MEK. We did not observe any statistically significant changes in body weight of mice used in the experiments (Figure 8C). Immunoblotting analyses were done to confirm the mechanisms of action of trametinib. Interestingly, P-ERK was increased in the knockdown STAT3 group, which is consistent with what observed in cell experiments (Figure 8D).

## DISCUSSION

Activating K-Ras mutations occur at a frequency of 90% in pancreatic and 45% in colorectal carcinomas. Currently, there have been no specific inhibitors for this oncogene [2]. Efforts to block oncogenic Ras activity are focused on downstream pathways. Inhibiting the downstream effector MEK1/2 has proven to be effective in preclinical and clinical studies in patients with melanoma, pancreatic, colon and lung cancers. So far, 11 MEK inhibitors have entered clinical trials. Among them, trametinib has been approved as cancer therapies [62]. Unfortunately, the clinical success of MEK inhibitors as single agents has often been limited by toxicity, low efficacy and drug resistance in K-Ras mutant cancers. Recently, more evidence has emerged to suggest that feedback activation of other pathway may limit the efficacy of MEK inhibitors in K-Ras mutated cancers [63]. Despite intensive study, the molecular and genetic mechanisms for drug resistance remain poorly understood. Preclinical studies have identified distinct mechanisms by which cells acquire resistance to MEK inhibition, including amplification of mutant BRAF [64], PI3K upregulation [23], EGFR activation [54] or mutations in the allosteric pocket of MEK, which can directly block the inhibitor binding to the MEK kinase or induce constitutive MEK kinase activity. Dual inhibition of these pathways has provided benefit in some patients [65]. In this study, we identified the JAK2/STAT3 pathway as a key mediator of the resistance to MEK inhibition in K-Ras mutant pancreatic and colon cancer cells.

The mechanism of STAT3 activation following MEK inhibitor treatment appeared complex. We initially identified that the MEK inhibitor AZD6244 stimulated phosphorylation of STAT3 mainly at Tyr705 residue. Since AZD6244 is not approved for cancer therapy, we then confirmed our observations with the FDA approved MEK selective inhibitor trametinib, which showed similar results of activating STAT3 mainly through Tyr705 phosphorylation. In tumors, where STAT3 was implicated for oncogenesis, activation of STAT3 was found to be the result of phosphorylation at both Ty705 and Ser727 residues. The role of STAT3 phosphorylation at Ty705 in tumorigenesis is well established. However, the function of phosphorylated Ser727 remains controversial at the moment. Our results indicate that MEK inhibition induced marked Tyr705 phosphorylation but only a slight Ser727 phosphorylation in the majority of K-Ras mutant cancer cell lines. The differences we found in Tyr705 and Ser727 phosphorylation of STAT3 are in line with the latter report [66], supporting the Tyr705 phosphorylation as an activating factor. The function of Ser727 phosphorylation may depend on the specific gene and cell type.

We further confirmed that inhibition of the STAT3 pathway by STAT3-specific shRNA or LY5 [67, 68] sensitized K-Ras mutated cancer cells to MEK inhibitor treatment *in vitro* and *in vivo*. In our study, stable cell lines expressing shRNA constructs against STAT3 in the K-Ras mutant AsPC-1 cell lines enhanced sensitivity to AZD6244 and trametinib, and activation of the STAT3 pathway with the constitutively active form of STAT3 induced resistance to AZD6244. Also, inhibiting the STAT3 pathway by LY5 enhances the ability of MEK inhibitors to inhibit cancer cell viability. Small molecule inhibitors that directly inhibit STAT3 are not yet approved by FDA for therapeutic use. However, compounds that inhibit upstream effectors of the STAT3 pathway, such as RTKs, Src and JAKs, are clinically available. Interestingly, our study shows that inhibiting Src or JAK3 does not cause extensive cell death compared with inhibiting STAT3 directly. In contrast, JAK2 inhibitor, ruxolitinib, significantly suppressed tumor cells viability in the K-Ras mutant cancer cells, which indicates that JAK2-STAT3 signaling contributes to the drug resistance in this model. Consistent with our findings, a combination of MEK and JAK2 inhibitors showed remarkable anti-cancer activity in these K-Ras mutant cancer cells [69, 70].

Our study indicates that inhibition of the single SAT3 or MEK pathway in the xenograft assay did not markedly reduce tumor growth, but dual inhibition of STAT3 and MEK pathways resulted in significant suppression of tumors, with subsequent slight tumor growth, and stop growing over the 5 weeks after treatment. The STAT3 shRNA tumors, however, showed a marked increase in phosphorylation of ERK after knockdown STAT3. The results suggested that ERK activation could compensate with the STAT3 inhibition to maintain proliferation and survival. This may also explain that knocking down STAT3 group alone did not reduce the size of xenograft tumors compared with AsPC-1(Vector) group.

## CONCLUSIONS

In conclusion, we explored novel compensatory mechanisms by which K-Ras mutant cells resist the MEK inhibitors. Our results provide the new evidence for the MEK inhibition can increase the expression and activation of STAT3 in K-Ras mutant pancreatic and colon cancer cells. Using genetic, functional, and pharmacologic inhibition, we have demonstrated that dual inhibition of STAT3 and MEK signaling pathways significantly reduced viability of pancreatic and colon cancer cells. Our study not only provides insight into the molecular mechanism of MEK inhibitor resistance but also suggests a novel combination therapy of STAT3 and MEK inhibitors could be a potential therapeutic strategy for preventing and/or overcoming resistance of MEK inhibitor in patients with colorectal and pancreatic cancers.

## MATERIALs AND METHODS

### Inhibitor drugs

Selumetinib (AZD6244, MEK inhibitor), trametinib (GSK1120212, MEK inhibitor), KX2-391 (Src inhibitor) and tofacitinib (CP-690550, JAK3 inhibitor) were purchased from Selleck Chemicals (Houston, TX, USA). PD98059 (MEK inhibitor) and ruxolitinib (INCB081424, JAK1/2 inhibitor) were purchased from LC Laboratories (Woburn, MA, USA). LY5 (STAT3 inhibitor [67]) was synthesized by Chenglong Li's Lab (College of Pharmacy, The Ohio State University). The drug compounds were dissolved in sterile dimethyl sulfoxide (DMSO) to make a 20 mM stock solution stored at −20°C.

### Cell culture

Human pancreatic cancer cell lines (PANC-1, HPAC and AsPC-1) and human colon cancer cell lines (HCT116, LS174T and DLD-1) were purchased from ATCC (the American Type Culture Collection, Manassas, VA, USA). All above cell lines were maintained in Dulbecco's Modified Eagle Medium (DMEM) supplemented with 10% fetal bovine serum (FBS) and 1% Penicillin/Streptomycin. The Capan-1 cell lines was provided by Dr. Mitch Phelps (The Ohio State University) and cultured in Iscove's Modified Dubecco's Medium (IMDM) supplemented with 20% FBS and 1% Penicillin/Streptomycin. All cell lines were cultured in a humidified 37 °C incubator with 5% CO_2_. Heat-inactivated fetal bovine serum (FBS), penicillin-streptomycin antibiotic, Dulbecco's Modified Eagle Medium (DMEM) containing 4.5g/L D-glucose, L-glutamine and sodium pyruvate, OptiMEM reduced serum medium and Iscove's Modified Dubecco's Medium (IMDM), both containing L-glutamine, were all purchased from Gibco (Grand Island, NY).

### Cell transfection

#### shRNA knockdown

The human STAT3 gene-specific shRNA plasmid, along with control shRNA plasmids were purchased from Addgene. Stable clones were prepared by transfecting AsPC-1 cells in 6-well dishes with 3 μg of each of the shRNA plasmids, using Lipofectamine 2000 transfection reagent (Invitrogen, Carlsbad, CA), according to manufacturer's instructions. Briefly, in each well of 6-well plates, 2.0 × 10^5^ AsPC-1 cells were grown in 2 mL DMEM culture medium without antibiotics to 50 - 70% confluence. Growth medium was replaced with 2 mL of freshly prepared Opti-MEM medium. Cells were then transfected with 3.0 μg of shRNA plasmid using 10 μL Lipofectamine 2000 Reagent. At 6 hours post-transfection, growth medium was replaced with a fresh complete DMEM medium with 10% FBS. 72 hours after transfection, the cells were placed under selection with 4.0μg/ml of puromycin, splitting 1:5 when the cells reached confluency. Clones from the transfected cells were isolated and grown under puromycin. Western blot assay was used to detect the expression of P-STAT3 and STAT3 in the cells.

#### STAT3-C transfection

The STAT3-C (a murine STAT3) was a constitutively active form of STAT3, which was cloned into vector with a Flag epitope [31]. AsPC-1 cells were plated in 6-well plate and grew to 70 - 90% confluence. Cells were then transfected with 3.0 μg of STAT3-C plasmid using 10 μL Lipofectamine 2000 Reagent in opti-MEM. After overnight transfection, growth medium was replaced with a fresh complete DMEM medium with 10% FBS. 48 hours after transfection, cells were split 1:5~1:50 into single cell. After 72 hours, transfected cells were placed under selection with 400μg/ml of G418. Clones from the transfected cells were isolated and grown under G418. Colones were confirmed by Western Blotting analysis. AsPC-1 cells expressing constitutive STAT3 were treated with AZD6244 (1–25μM) or DMSO. Cell viability was determined by MTT assay in 96-well plates as described in this paper.

### MTT cell viability assay

Cells were seeded in 96-well plates at a density of 3000 cells per well and cultured for 24 hours. For cell viability experiment, different concentrations of drugs were added in triplicate to the plates for 24-72 hours at 37 °C. 3-(4, 5-Dimethylthiazolyl)-2, 5-diphenyltetrazolium bromide (MTT) viability assay was done according to manufacturer's protocol (Roche Diagnostics, Mannheim, Germany). The absorbance was read at 595 nm. Half-maximal inhibitory concentrations (IC_50_) were determined using Sigma Plot 9.0 software (Systat Software Inc., San Jose, CA). Each experiment was repeated 3 times. Data are given as mean ± SD.

### Western blot analysis

Cells were harvested after treatment with drugs or DMSO at 60-80% confluence for 24-72 hours then lysed in cold RIPA lysis buffer containing a cocktail of protease inhibitors and phosphatase inhibitor. The lysates were subjected to 10% or 12% SDS-PAGE gel and transferred to a PVDF membrane. Membranes were incubated with a 1:1000 dilution of specific primary antibody and 1:10000 HRP conjugated secondary antibody. Primary antibodies including phospho-STAT3 (Tyrosine 705), phospho-STAT3 (Ser727), STAT3, phospho-AKT (Serine 473), phospho-ERK1/2 (Threonine 202/Tyrosine 204), phospho-JAK2(Tyr1007/1008), phospho-JAK3 (Tyr980/981), phospho-Src (Tyrosine 416) and GAPDH, and secondary antibody are all from Cell Signaling Technology (Beverly, MA, USA). Membranes were analyzed using enhanced chemiluminescence Plus reagents and scanned with the Storm Scanner (Amersham Pharmacia Biotech Inc, Piscataway, NJ).

### Colony formation assay

Cells were grown to 60–80% confluent in 10 cm plates and then pre-treated with drugs or DMSO for 2-6 hours. Then the treated cells were trypsinized, stained with trypan blue and counted. 1000 cells were then seeded in 60 mm plates in duplicate and allowed to grow for 2-3 weeks until the colonies were visible. Cells were washed with PBS twice and fixed with cold methanol at −20 °C for 10 min. Cells were then stained with 1% crystal violet (25% methanol) at room temperature for 10 min. After the staining, the plates were washed with distill water and dried.

### Cell migration assay (in vitro wound-healing assay)

HPAC, DLD-1 and HCT-116 cells migration were detected using the wound-healing assay described by Xiao et al. [68]. Briefly, when cells grew into confluent monolayer in plate, we scratched the cells in same width using yellow tip and washed once to remove non-adherent cells. After washing, cells treated with LY5 (2μΜ/1μΜ), trametinib (1μM) or DMSO. Cells were allowed to migrate into scratched area for 24-72 hours and images were captured with a microscopic camera system. The percentage of wound healing was calculated by the equation: (percent wound healing) = average of [(gap area: 0 hour) − (gap area: 24-72 hours)]/ (gap area: 0 hours) [68].

### Animal studies

All animal studies were conducted in accordance with the standard procedures approved by the Institutional Animal Care and Use Committee (IACUC) of the Research Institute at Nationwide Children's Hospital (Columbus, OH). The approved protocols were designed to minimize the numbers of mice used and to minimize any pain or distress. Mice were acclimatized at the Animal Facility for 1 week before being injected with cancer cells. For analysis of tumorigenicity, AsPC-1(vector) control or AsPC-1 STAT3 shRNA cells (5×10^6^ cells/mice) were mixed with an equal volume of PBS and Matrigel (Invitrogen) in a total of 100 μL and were injected subcutaneously into the flank of 6-week-old, weighing 20 to 25 g, athymic female nude mice. All mice were maintained under barrier conditions. After 1 week, tumor mass reaching 6 mm in diameter was judged as a successful model and mice were randomly divided into four groups (n=4). Drug treated group of animals were given MEK inhibitor trametinib 0.30mg/kg/day [drug in 5%DMSO, 10%Kolliphor^®^HS15 and 85% (20% (2-Hydroxypropyl)-β-cyclodextrin)] by daily oral gavage. The first day of drug treatment was termed day 0. The maximum diameter (a) and minimum diameter (b) of the tumor were measured with a vernier caliper every 2 days, and at the same time, the behavior, diet, and weight of nude mice were observed. The tumor volume (V) was calculated according to the formula: volume = (π/6) ab^2^. At the end of treatments, tumors were harvested from euthanized mice, snap-frozen in liquid nitrogen and stored in −80°C. Tumors tissue homogenates were lysed and separated by SDS-PAGE to examine the expression of P-ERK and P-STAT3.

### Statistical analysis

Significance of correlations was done using GraphPad Prism software. Unpaired t tests were used for analyses assuming Gaussian populations with a 95% confidence interval. Data are presented as mean ± SD. Differences were analyzed with the Student t test, and significance was set at *P* < 0.05.

## References

[1] Saif MW, Shah M (2009). K-ras mutations in colorectal cancer: a practice changing discovery. Clin Adv Hematol Oncol.

[2] Van Der Hoeven D, Cho KJ, Ma X, Chigurupati S, Parton RG, Hancock JF (2013). Fendiline inhibits K-Ras plasma membrane localization and blocks K-Ras signal transmission. Mol Cell Biol.

[3] Forrester K, Almoguera C, Han KY, Grizzle WE, Perucho M (1987). Detection of High-Incidence of K-Ras Oncogenes during Human-Colon Tumorigenesis. Nature.

[4] Moon BS, Jeong WJ, Park J, Kim TI, Min DS, Choi KY (2014). Role of Oncogenic K-Ras in Cancer Stem Cell Activation by Aberrant Wnt/beta-Catenin Signaling. J Natl Cancer Inst.

[5] Smit VTHBM, Boot AJM, Smits AMM, Fleuren GJ, Cornelisse CJ, Bos JL (1988). Kras Codon-12 Mutations Occur Very Frequently in Pancreatic Adenocarcinomas. Nucleic Acids Res.

[6] Almoguera C, Shibata D, Forrester K, Martin J, Arnheim N, Perucho M (1988). Most Human Carcinomas of the Exocrine Pancreas Contain Mutant C-K-Ras. Genes Cell.

[7] Wei S, Liang Z, Gao J, Wu S, Zhu H, Liu H, Liu T (2005). Patterns of K-ras codon 12 and 13 mutations found in pancreatic adenocarcinoma of 30 Chinese patients by microdissection, PCR and direct sequencing. J Gastroen Hepatol.

[8] Pellegata NS, Sessa F, Renault B, Bonato M, Leone BE, Solcia E, Ranzani GN (1994). K-Ras and P53 Gene-Mutations in Pancreatic-Cancer-Ductal and Nonductal Tumors Progress through Different Genetic Lesions. Cancer Res.

[9] Matos P, Oliveira C, Velho S, Gonçalves V, da Costa LT, Moyer MP, Seruca R, Jordan P (2008). B-Raf(V600E) cooperates with alternative spliced Rac1b to sustain colorectal cancer cell survival. Gastroenterology.

[10] Sasaki M, Sugio K, Sasazuki T (1990). K-Ras Activation in Colorectal Tumors from Patients with Familial Polyposis-Coli. Cancer.

[11] Bos JL (1989). Ras Oncogenes in Human Cancer-a Review. Cancer Res.

[12] Vaughn CP, ZoBell SD, Furtado LV, Baker CL, Samowitz WS (2011). Frequency of KRAS, BRAF, and NRAS Mutations in Colorectal Cancer. Gene Chromosome Canc.

[13] Pollock CB, Shirasawa S, Sasazuki T, Kolch W, Dhillon AS (2005). Oncogenic K-RAS is required to maintain changes in cytoskeletal organization, adhesion, and motility in colon cancer cells. Cancer Res.

[14] Shima F, Yoshikawa Y, Ye M, Araki M, Matsumoto S, Liao J, Hu L, Sugimoto T, Ijiri Y, Takeda A, Nishiyama Y, Sato C, Muraoka S (2013). In silico discovery of small-molecule Ras inhibitors that display antitumor activity by blocking the Ras-effector interaction. P Natl Acad Sci USA.

[15] Ostrem JM, Peters U, Sos ML, Wells JA, Shokat KM (2013). K-Ras(G12C) inhibitors allosterically control GTP affinity and effector interactions. Nature.

[16] Young A, Lyons J, Miller AL, Phan VT, Alarcon IR, McCormick F (2009). Ras signaling and therapies. Adv Cancer Res.

[17] Surade S, Blundell TL (2012). Structural biology and drug discovery of difficult targets: the limits of ligandability. Chem Biol.

[18] Chan DA, Giaccia AJ (2011). Harnessing synthetic lethal interactions in anticancer drug discovery. Nat Rev Drug Discov.

[19] Corcoran RB, Contino G, Deshpande V, Tzatsos A, Conrad C, Benes CH, Levy DE, Settleman J, Engelman JA, Bardeesy N (2011). STAT3 plays a critical role in KRAS-induced pancreatic tumorigenesis. Cancer Res.

[20] Downward J (2003). Targeting RAS signalling pathways in cancer therapy. Nat Rev Cancer.

[21] Campbell PM, Groehler AL, Lee KM, Ouellette MM, Khazak V, Der CJ (2007). K-Ras promotes growth transformation and invasion of immortalized human pancreatic cells by Raf and phosphatidylinositol 3-kinase signaling. Cancer Res.

[22] Engelman JA, Chen L, Tan X, Crosby K, Guimaraes AR, Upadhyay R, Maira M, McNamara K, Perera SA, Song Y, Chirieac LR, Kaur R, Lightbown A (2008). Effective use of PI3K and MEK inhibitors to treat mutant Kras G12D and PIK3CA H1047R murine lung cancers. Nat Med.

[23] Sos ML, Fischer S, Ullrich R, Peifer M, Heuckmann JM, Koker M, Heynck S, Stückrath I, Weiss J, Fischer F, Michel K, Goel A, Regales L (2009). Identifying genotype-dependent efficacy of single and combined PI3K-and MAPK-pathway inhibition in cancer. P Natl Acad Sci USA.

[24] Rinehart J, Adjei AA, Lorusso PM, Waterhouse D, Hecht JR, Natale RB, Hamid O, Varterasian M, Asbury P, Kaldjian EP, Gulyas S, Mitchell DY, Herrera R (2004). Multicenter phase II study of the oral MEK inhibitor, CI-1040, in patients with advanced non-small-cell lung, breast, colon, and pancreatic cancer. J Clin Oncol.

[25] Watanabe M, Sowa Y, Yogosawa M, Sakai T (2013). Novel MEK inhibitor trametinib and other retinoblastoma gene (RB)-reactivating agents enhance efficacy of 5-fluorouracil on human colon cancer cells. Cancer Sci.

[26] Jing J, Greshock J, Holbrook JD, Gilmartin A, Zhang X, McNeil E, Conway T, Moy C, Laquerre S, Bachman K, Wooster R, Degenhardt Y (2012). Comprehensive Predictive Biomarker Analysis for MEK Inhibitor GSK1120212. Mol Cancer Ther.

[27] Neuzillet C, Hammel P, Tijeras-Raballand A, Couvelard A, Raymond E (2013). Targeting the Ras-ERK pathway in pancreatic adenocarcinoma. Cancer Metast Rev.

[28] Bodoky G, Timcheva C, Spigel DR, La Stella PJ, Ciuleanu TE, Pover G, Tebbutt NC (2012). A phase II open-label randomized study to assess the efficacy and safety of selumetinib (AZD6244 [ARRY-142886]) versus capecitabine in patients with advanced or metastatic pancreatic cancer who have failed first-line gemcitabine therapy. Invest New Drug.

[29] Buettner R, Mora LB, Jove R (2002). Activated STAT signaling in human tumors provides novel molecular targets for therapeutic intervention. Clin Cancer Res.

[30] Turkson J, Jove R (2000). STAT proteins: novel molecular targets for cancer drug discovery. Oncogene.

[31] Bromberg JF, Wrzeszczynska MH, Devgan G, Zhao Y, Pestell RG, Albanese C, Darnell JE (1999). Stat3 as an oncogene. Cell.

[32] Inghirami G, Chiarle R, Simmons WJ, Piva R, Schlessinger K, Levy DE (2005). New and old functions of STAT3-A pivotal target for individualized treatment of cancer. Cell Cycle.

[33] Schlessinger K, Levy DE (2005). Malignant transformation but not normal cell growth depends on signal transducer and activator of transcription 3. Cancer Res.

[34] Scholz A, Heinze S, Detjen KM, Peters M, Welzel M, Hauff P, Schirner M, Wiedenmann B, Rosewicz S (2003). Activated signal transducer and activator of transcription 3 (STAT3) supports the malignant phenotype of human pancreatic cancer. Gastroenterology.

[35] Wei D, Le X, Zheng L, Wang L, Frey JA, Gao AC, Peng Z, Huang S, Xiong HQ, Abbruzzese JL, Xie K (2003). Stat3 activation regulates the expression of vascular endothelial growth factor and human pancreatic cancer angiogenesis and metastasis. Oncogene.

[36] Corvinus FM, Orth C, Moriggl R, Tsareva SA, Wagner S, Pfitzner EB, Baus D, Kaufmann R, Huber LA, Zatloukal K, Beug H, Ohlschläger P, Schütz A (2005). Persistent STAT3 activation in colon cancer is associated with enhanced cell proliferation and tumor growth. Neoplasia.

[37] Lin L, Liu A, Peng Z, Lin HJ, Li PK, Li C, Lin J (2011). STAT3 is Necessary for Proliferation and Survival in Colon Cancer-Initiating Cells. Cancer Res.

[38] Lin Q, Lai R, Chirieac LR, Li C, Thomazy VA, Grammatikakis I, Rassidakis GZ, Zhang W, Fujio Y, Kunisada K, Hamilton SR, Amin HM (2005). Constitutive activation of JAK3/STAT3 in colon carcinoma tumors and cell lines-Inhibition of JAK3/STAT3 signaling induces apoptosis and cell cycle arrest of colon carcinoma cells. Am J Pathol.

[39] Klampfer L (2008). The role of signal transducers and activators of transcription in colon cancer. Front Biosci-Landmrk.

[40] Lassmann S, Schuster I, Walch A, Göbel H, Jütting U, Makowiec F, Hopt U, Werner M (2007). STAT3 mRNA and protein expression in colorectal cancer: effects on STAT3-inducible targets linked to cell survival and proliferation. J Clin Pathol.

[41] Kusaba T, Nakayama T, Yamazumi K, Yakata Y, Yoshizaki A, Nagayasu T, Sekine I (2005). Expression of p-STAT3 in human colorectal adenocarcinoma and adenoma; correlation with clinicopathological factors. J Clin Pathol.

[42] Lee J Y, Hennighausen L (2005). The transcription factor Stat3 is dispensable for pancreatic beta-cell development and function. Biochem Bioph Res Co.

[43] Kusaba T, Nakayama T, Yamazumi K, Yakata Y, Yoshizaki A, Inoue K, Nagayasu T, Sekine I (2006). Activation of STAT3 is a marker of poor prognosis in human colorectal cancer. Oncol Rep.

[44] Huang C, Cao J, Huang KJ, Zhang F, Jiang T, Zhu L, Qiu ZJ (2006). Inhibition of STAT3 activity with AG490 decreases the invasion of human pancreatic cancer cells in vitro. Cancer Sci.

[45] Lin L, Hutzen B, Zuo M, Ball S, Deangelis S, Foust E, Pandit B, Ihnat MA, Shenoy SS, Kulp S, Li PK, Li C, Fuchs J (2010). Novel STAT3 Phosphorylation Inhibitors Exhibit Potent Growth-Suppressive Activity in Pancreatic and Breast Cancer Cells. Cancer Res.

[46] Glienke W, Hausmann E, Bergmann L (2011). Downregulation of STAT3 signaling induces apoptosis but also promotes anti-apoptotic gene expression in human pancreatic cancer cell lines. Tumor Biol.

[47] Lin L, Hutzen B, Li PK, Ball S, Zuo M, DeAngelis S, Foust E, Sobo M, Friedman L, Bhasin D, Cen L, Li C, Lin J (2010). A Novel Small Molecule, LLL12, Inhibits STAT3 Phosphorylation and Activities and Exhibits Potent Growth-Suppressive Activity in Human Cancer Cells. Neoplasia.

[48] Thoennissen NH, Iwanski GB, Doan NB, Okamoto R, Lin P, Abbassi S, Song JH, Yin D, Toh M, Xie WD, Said JW, Koeffler HP (2009). Cucurbitacin B Induces Apoptosis by Inhibition of the JAK/STAT Pathway and Potentiates Antiproliferative Effects of Gemcitabine on Pancreatic Cancer Cells. Cancer Res.

[49] Fan Y, Zhang YL, Wu Y, Zhang W, Wang YH, Cheng ZM, Li H (2008). Inhibition of signal transducer and activator of transcription 3 expression by RNA interference suppresses invasion through inducing anoikis in human colon cancer cells. World J Gastroentero.

[50] Metro G, Chiari R, Baldi A, De Angelis V, Minotti V, Crino L (2013). Selumetinib: a promising pharmacologic approach for KRAS-mutant advanced non-small-cell lung cancer. Future Oncol.

[51] Alessi DR, Cuenda A, Cohen P, Dudley DT, Saltiel AR (1995). PD 098059 is a specific inhibitor of the activation of mitogen-activated protein kinase kinase in vitro and in vivo. J Biol Chem.

[52] Kim KB, Kefford R, Pavlick AC, Infante JR, Ribas A, Sosman JA, Fecher LA, Millward M, McArthur GA, Hwu P, Gonzalez R, Ott PA, Long GV (2013). Phase II Study of the MEK1/MEK2 Inhibitor Trametinib in Patients With Metastatic BRAF-Mutant Cutaneous Melanoma Previously Treated With or Without a BRAF Inhibitor. J Clin Oncol.

[53] Flaherty KT, Robert C, Hersey P, Nathan P, Garbe C, Milhem M, Demidov LV, Hassel JC, Rutkowski P, Mohr P, Dummer R, Trefzer U, Larkin JM (2012). Improved Survival with MEK Inhibition in BRAF-Mutated Melanoma. New Engl J Med.

[54] Girotti MR, Pedersen M, Sanchez-Laorden B, Viros A, Turajlic S, Niculescu-Duvaz D, Zambon A, Sinclair J, Hayes A, Gore M, Lorigan P, Springer C, Larkin J (2013). Inhibiting EGF receptor or SRC family kinase signaling overcomes BRAF inhibitor resistance in melanoma. Cancer Discov.

[55] Hirano T, Ishihara K, Hibi M (2000). Roles of STAT3 in mediating the cell growth, differentiation and survival signals relayed through the IL-6 family of cytokine receptors. Oncogene.

[56] McCubrey JA, Steelman LS, Abrams SL, Chappell WH, Russo S, Ove R, Milella M, Tafuri A, Lunghi P, Bonati A, Stivala F, Nicoletti F, Libra M (2010). Emerging MEK inhibitors. Expert Opin Emerg Drugs.

[57] Chappell WH, Steelman LS, Long JM, Kempf RC, Abrams SL, Franklin RA, Bäsecke J, Stivala F, Donia M, Fagone P, Malaponte G, Mazzarino MC, Nicoletti F (2011). Ras/Raf/MEK/ERK and PI3K/PTEN/Akt/mTOR Inhibitors: Rationale and Importance to Inhibiting These Pathways in Human Health. Oncotarget.

[58] Diep CH, Munoz RM, Choudhary A, Von Hoff DD, Han HY (2011). Synergistic Effect between Erlotinib and MEK Inhibitors in KRAS Wild-Type Human Pancreatic Cancer Cells. Clin Cancer Res.

[59] Grivennikov S1, Karin E, Terzic J, Mucida D, Yu GY, Vallabhapurapu S, Scheller J, Rose-John S, Cheroutre H, Eckmann L, Karin M (2009). IL-6 and Stat3 are required for survival of intestinal epithelial cells and development of colitis-associated cancer. Cancer cell.

[60] Kanda N, Seno H, Konda Y, Marusawa H, Kanai M, Nakajima T, Kawashima T, Nanakin A, Sawabu T, Uenoyama Y, Sekikawa A, Kawada M, Suzuki K (2004). STAT3 is constitutively activated and supports cell survival in association with survivin expression in gastric cancer cells. Oncogene.

[61] Aoki Y, Feldman GM, Tosato G (2003). Inhibition of STAT3 signaling induces apoptosis and decreases survivin expression in primary effusion lymphoma. Blood.

[62] Akinleye A, Furqan M, Mukhi N, Ravella P, Liu D (2013). MEK and the inhibitors: from bench to bedside. J Hematol Oncol.

[63] Zhao Y, Adjei AA (2014). The clinical development of MEK inhibitors, Nature reviews. Clinical oncology.

[64] Little AS, Balmanno K, Sale MJ, Newman S, Dry JR, Hampson M, Edwards PA, Smith PD, Cook SJ (2011). Amplification of the Driving Oncogene, KRAS or BRAF, Underpins Acquired Resistance to MEK1/2 Inhibitors in Colorectal Cancer Cells. Sci Signal.

[65] Alagesan B, Contino G, Guimaraes AR, Corcoran RB, Deshpande V, Wojtkiewicz GR, Hezel AF, Wong KK, Loda M, Weissleder R, Benes C, Engelman JA, Bardeesy N (2014). Combined MEK and PI3K inhibition in a mouse model of pancreatic cancer. Clin Cancer Res.

[66] Huang G, Yan H, Ye S, Tong C, Ying QL (2014). STAT3 phosphorylation at tyrosine 705 and serine 727 differentially regulates mouse ESC fates. Stem Cells.

[67] Yu W, Xiao H, Lin J, Li C (2013). Discovery of novel STAT3 small molecule inhibitors via in silico site-directed fragment-based drug design. J Med Chem.

[68] Xiao H, Bid HK, Jou D, Wu X, Yu W, Li C, Houghton PJ, Lin J (2015). A Novel Small Molecular STAT3 Inhibitor, LY5 Inhibits Cell Viability, Cell Migration, And Angiogenesis in Medulloblastoma Cells. J Biol Chem.

[69] Lee HJ, Zhuang G, Cao Y, Du P, Kim HJ, Settleman J (2014). Drug resistance via feedback activation of Stat3 in oncogene-addicted cancer cells. Cancer cell.

[70] Van Schaeybroeck S, Kalimutho M, Dunne PD, Carson R, Allen W, Jithesh PV, Redmond KL, Sasazuki T, Shirasawa S, Blayney J, Michieli P, Fenning C, Lenz HJ (2014). ADAM17-dependent c-MET-STAT3 signaling mediates resistance to MEK inhibitors in KRAS mutant colorectal cancer. Cell Rep.

